# Transcriptional Heterogeneity of Cardiac Remodeling Between Type 1 and Type 2 Diabetes

**DOI:** 10.3390/biomedicines14040746

**Published:** 2026-03-25

**Authors:** Feng Liang, Shaohua Li, Guo Zhou, Huanhuan Huo, Yijie Huang, Haiping Chen, Zhaohua Cai, Yi Li, Ben He

**Affiliations:** 1 Department of Cardiology, Shanghai Chest Hospital, Shanghai Jiao Tong University School of Medicine, Shanghai 200030, China; 2 Department of Pharmacy, Zhongshan Hospital, Fudan University, Shanghai 200032, China; 3 Shanghai Pudong New Area Pulmonary Hospital, Shanghai 200000, China; 4 New Cornerstone Investigator Institute, State Key Laboratory of Cell Biology, Shanghai Institute of Biochemistry and Cell Biology, Center for Excellence in Molecular Cell Science, Chinese Academy of Sciences, University of Chinese Academy of Sciences, Shanghai 200234, China; 5 Department of Cardiology, Shanghai East Hospital, Tongji University School of Medicine, Shanghai 200120, China

**Keywords:** type 1 diabetes, type 2 diabetes, multi-omics, heterogeneity

## Abstract

**Background:** Cardiovascular complications stemming from diabetes pose a grave threat to patients’ survival. Both type 1 diabetes (T1D) and type 2 diabetes (T2D) significantly increase the risk of heart failure, yet no reports have clarified whether there are differences in the pathway alterations involved in these two conditions. Investigating the heterogeneity of the cardiac remodeling between these two types of diabetes is conducive to reducing the incidence of cardiovascular events in diabetic patients in clinical practice. **Methods:** T1D and T2D models were established in adult mice, and the hearts were collected for RNA sequencing. Differential expression analysis (DEA) was performed. Integrating functional enrichment analyses, we probed into gene and pathway heterogeneity. Subsequently, we compared single-cell RNA sequencing (scRNA-seq) data of hearts from T1D and T2D mice, focusing on three cell populations (endothelial cells, macrophages, and fibroblasts) to identify gene and pathway differences. Finally, we evaluated shared genes and common signaling pathway changes across these three cell populations in both diabetes types. **Results:** We have successfully established T1D and T2D models in mice. Compared with shared genes, the two types of diabetes had more consistent pathway changes. Further scRNA-seq analysis identified endothelial cells, macrophages, and fibroblasts as significantly associated with the diabetic phenotype. In shared pathway, endothelial cells were significantly enriched in pathways related to endothelial proliferation and angiogenesis; macrophages were enriched in immune response pathways; and fibroblasts were enriched in pathways involving fibrosis, cell proliferation, and apoptosis. In endothelial cells, inflammatory response and fatty acid metabolism pathways were predominantly enriched in T1D, while energy metabolism pathways were dominant in T2D. In macrophages, antiviral immune pathways were specifically enriched in T1D, whereas macrophages in T2D were additionally implicated in the regulation of cardiomyocyte function. In fibroblasts, immune-related pathways were characteristically enriched in T1D, while cell respiration and energy supply pathways were prominent in T2D. Common functional enrichment pathways across the three cell types in both diabetes types mainly involved innate immune responses and cardiac morphogenesis, with the proportion of shared pathways being significantly higher than that of shared genes. **Conclusions:** This study, by combining RNA sequencing and scRNA-seq, revealed that cardiac pathologies induced by T1D and T2D exhibit a higher degree of consistent pathway changes compared to shared gene changes. Interventions targeting these common pathways may hold greater value in preventing and treating diabetic cardiomyopathy.

## 1. Background

Diabetes is one of the leading causes of death and disability globally, exerting an immense burden on healthcare systems, economies, and societies at large. In 2021, the global diabetic population reached 529 million, corresponding to an age-standardized prevalence of 6.1% [1]. It is projected that the global prevalence of diabetes will rise to 9.8% by 2050 [1]. T1D and T2D are the two most prominent forms, with T2D accounting for over 96% of all cases [1]. T1D results from immune-mediated β-cell destruction, leading to insulin deficiency. In contrast, T2D is a heterogeneous disorder characterized by varying degrees of β-cell dysfunction combined with insulin resistance [2,3]. Both types predispose patients to severe macrovascular and microvascular complications, which are major contributors to mortality and disability. A Swedish cohort study demonstrated that T1D increases the risk of cardiovascular diseases by more than 4-fold, with a more pronounced effect in women [4]. By contrast, patients with T2D carry a 2–3-fold higher risk of cardiovascular disease than the general population, among which the excess risk of heart failure is particularly prominent [5,6]. These heart complications caused by diabetes significantly shorten the human lifespan [2,7].

Diabetic cardiomyopathy (DCM) refers to a specific cardiac disorder that develops in diabetic patients in the absence of coronary artery disease, hypertension, or valvular heart disease. It is structurally and functionally featured by ventricular dysfunction and gradual progression toward heart failure, which are mainly driven by chronic metabolic disorders, including abnormal glucose homeostasis and dysregulated lipid metabolism [8]. DCM features are characterized by cardiac remodeling, fibrosis, and endothelial dysfunction, which ultimately progress to heart failure [9,10]. Cardiac remodeling in T1D and T2D share common pathophysiological features, mainly including cardiac hypertrophy, interstitial fibrosis, cardiomyocyte apoptosis, and diastolic and/or systolic myocardial dysfunction [11]. The shared pathogenic mechanisms involve hyperglycemia, lipotoxicity, inflammation and oxidative stress, microvascular dysfunction, and cardiac autonomic neuropathy [12]. The unique pathological mechanism of type 1 diabetes is cardiac autoimmunity and decreased autophagy, whereas type 2 diabetes is characterized by hyperinsulinemia and aberrant autophagy. Collectively, these mechanisms ultimately lead to similar structural and functional alterations in the heart, such as fibrosis and myocardial dysfunction [13]. As an important glycemic regulator, reported circulating glucagon-like peptide-1 (GLP-1) levels in diabetic patients remain inconsistent. Several studies showed no significant differences in fasting or postprandial GLP-1 levels between T2D patients and controls, though results may be affected by treatment status and age [14,15]. A Chinese study reported elevated GLP-1 levels in young T2D patients (<60 years) but reduced levels in elderly patients [16]. In T1D, GLP-1 levels were either unchanged or decreased relative to controls [17,18]. The homogeneity and heterogeneity in cardiac remodeling induced by T1D and T2D, together with their distinct molecular and signaling mechanisms, remain to be fully elucidated.

With the continuous innovation of research technologies, especially the application of single-cell RNA sequencing (scRNA-seq) technology, it has become more convenient and accurate to explore the cellular and molecular mechanisms of cardiac remodeling in diabetes. In the diabetic pathological state, studies have reported that fatty acid metabolism in cardiomyocytes is enhanced, accompanied by a decline in myocardial contractile function [19]. Abnormal cytosolic Ca^2+^ handling in cardiomyocytes has been recognized as a key contributor to the development of DCM in both T1D and T2D [20]. In T1D mouse models, impaired sarcoplasmic reticulum function is closely associated with markedly reduced expression of sarcoplasmic/endoplasmic reticulum Ca^2+^ ATPase 2a (SERCA2a), leading to decreased contractile function of cardiomyocytes [21]. In T2D mice, cardiomyocytes similarly exhibit impaired contractile function, and diastolic dysfunction, resulting from downregulated expression of SERCA2a and RyR2 receptors, and subsequent disruption of intracellular calcium homeostasis [22]. Fibroblasts, by contrast, become activated to regulate myocardial function and the progression of fibrosis, while endothelial cell dysfunction and microcirculatory disorders occur concurrently [19]. Diabetes-induced cardiac fibrosis is ultimately mediated by cardiac fibroblasts. Hyperglycemia directly activates cardiac fibroblasts, increases oxidative stress, stimulates neurohumoral cascades, induces and activates various growth factors and cytokines, and promotes the accumulation of advanced glycation end products (AGEs). AGEs cross-link interstitial extracellular matrix (ECM) proteins and further aggravate cardiac fibrosis [23]. Additionally, pro-inflammatory monocytes increase, whereas reparative macrophages decrease, thus triggering inflammatory responses and upregulating proinflammatory cytokines to mobilize immune reactions [24,25]. Further studies combined with spatial transcriptomics have shown that in the coronary microvascular enrichment area of T2D, fatty acid metabolism and oxidative phosphorylation are significantly upregulated, revealing the specific characteristics of molecular expression in this area [26]. Although existing studies have initially clarified some cellular and molecular mechanisms of diabetic cardiac remodeling, there is still a lack of systematic and in-depth research on the specific differences in molecular pathways and pathological characteristics of cardiac remodeling between the two main types of diabetes, thereby defining the key direction and core demand for subsequent research.

Despite the high incidence of diabetic cardiac complications and extensive basic research, studies exploring the similarities and differences between T1D- and T2D-induced cardiac pathologies remain limited. Medical advancements have significantly prolonged the lifespan of diabetic patients. However, direct comparison between the two types of diabetes is challenging due to differences in their baseline characteristics and disease duration. The identification of occult T1D [27], early onset T2D [28], and double diabetes [29] has further complicated their distinction. Patients with T2D lose approximately 2–5% of their pancreatic β-cell function annually, and some patients with a disease duration of more than 20 years exhibit complete loss of islet function [30,31]. We therefore hypothesized that exploring shared pathological mechanisms underlying diabetic cardiac remodeling may be more valuable. In this study, we established T1D and T2D mouse models, profiled transcriptional differences in cardiac pathologies using transcriptome sequencing, and validated these differences and commonalities with scRNA-seq data. This study aims to provide new insights for clinical management and basic research for the prevention and management of diabetic cardiac complications.

## 2. Methods

### 2.1. Animal Model Construction

T1D model: Eight-week-old adult male C57BL/6J mice were purchased from Shanghai Model Organisms Center, Inc., (Shanghai, China) with 5 mice per group. Mice were housed in a specific-pathogen-free (SPF) facility with a controlled temperature of 22 ± 2 °C, a 12 h light/12 h dark cycle, and free access to standard chow and water. Animals were housed in groups of 3–5 mice per cage throughout the experiment. All mice were weighed and selected within a body weight range of 25 ± 1 g. Baseline blood glucose was measured to confirm normoglycemia prior to random grouping. The diabetic model was established by intraperitoneal injection of streptozotocin (STZ; S0130, Sigma -Aldrich (Shanghai) Trading Co. Shanghai, China) at 50 mg/kg for 5 consecutive days, while control mice received an equivalent volume of citrate buffer [32]. Fasting and random blood glucose levels were monitored; the model was considered successful if fasting blood glucose (after 8 h of fasting) exceeded 11.1 mmol/L. Mice were euthanized 8 weeks after model establishment for subsequent tissue collection.

T2D model: Eight-week-old adult male C57BL/6J mice were used, with 5 mice per group, same as above. Animals were weighed and selected within a body weight range of 25 ± 1 g. Baseline blood glucose was measured to confirm normoglycemia prior to randomization. Mice were randomly assigned to two groups (*n* = 5 per group): the experimental group was fed a high-fat diet (D12492, Research Diets), while the control group received a standard chow diet. After 12 weeks, the high-fat diet group was intraperitoneally injected with STZ at 30 mg/kg for 7 consecutive days [33], and the control group received an equivalent volume of citrate buffer. Successful diabetic model establishment was defined as fasting blood glucose > 11.1 mmol/L after 8 h of fasting. Mice were maintained on their respective diets for an additional 8 weeks before tissue collection.

All animal experiments were performed in accordance with the institutional guidelines for laboratory animal care and use and approved by the Institutional Animal Care and Use Committee (IACUC) of Shanghai Chest Hospital (Approval No. KS23042). The baseline characteristics of mouse model are attached as Supplementary Table S1. The composition and calorie content of each component in the high-fat diet are detailed in Supplementary Table S2.

### 2.2. Oral Glucose Tolerance Test

The oral glucose tolerance test (OGTT) was performed as previously described [34]. Briefly, mice were fasted for 8 h and then administered 2 g/kg glucose solution orally. Blood glucose levels were measured at 0, 30, 60, 90, and 120 min using a glucometer (Accu-Chek Guide Me, Roche Diagnostics, Roche Diabetes Care GmbH, Raunheim, Germany). For glucose tolerance analysis, the total area under the curve (total AUC) was calculated for each mouse in group.

### 2.3. Cardiac RNA Extraction and Transcriptome Sequencing

RNA extraction was performed as previously described [35]. Whole hearts fromT1D, T2D, and matched control mice (4 biological replicates per group) were lysed in 1 mL TRIzol (Invitrogen, Cat# 15596018, Carlsbad, CA, USA) for 10 min. Chloroform (200 μL) was added, and samples were vortexed vigorously for 15 s and incubated for 5 min. Samples were centrifuged at 12,000× *g* for 15 min at 4 °C. The upper aqueous phase was transferred, mixed with an equal volume of isopropanol, incubated for 10 min, and centrifuged at 12,000× *g* for 15 min at 4 °C. The RNA pellet was washed three times with 75% ethanol, air-dried at room temperature, and dissolved in RNase-free water. Total RNA was quantified and qualified using a NanoDrop (Thermo Fisher Scientific, Waltham, MA, USA) and Agilent 2100 Bioanalyzer (Thermo Fisher Scientific, Waltham, MA, USA).

High-quality RNA was used for mRNA library construction. mRNA was amplified using oligo-dT and dNTPs, followed by cDNA reverse transcription targeting polyA tails. cDNA was purified and size-selected using the Agencourt AMPure XP-Medium Kit (Thermo Fisher Scientific, Waltham, MA, USA) and quantified with the Agilent Technologies 2100 Bioanalyzer. Double-stranded PCR products were heat-denatured and circularized via splint oligonucleotide sequences to form single-stranded circular DNA as the final library. Sequencing was performed on the Illumina PE150 platform. Raw sequencing data were aligned to the Ensembl mouse genome (mm10, GRCm38) using Hisat2 (v0.1.6-beta) with default parameters. Reads aligned to genes were counted using HTSeq (v2.0.1), including only reads mapping to exonic regions and aggregating counts per gene. Reads overlapping multiple genes or mapping to multiple regions were excluded to generate the final gene expression matrix. Quality control parameters for RNA sequencing results are presented in Supplementary Table S3.

### 2.4. Differential Expression Analysis and Enrichment Analysis

For DEA in T1D and T2D mice, count matrices from upstream processing were analyzed using the DESeq2 package [36]. Steps included constructing a DESeq2 dataset, differential analysis based on negative binomial distribution, and result extraction. Differentially expressed genes (DEGs) between diabetic groups and controls were filtered using thresholds adjusted *p*-value < 0.05 and |FoldChange| > 1.5. Volcano plots were generated to visualize upregulated and downregulated DEGs.

To explore potential biological functions in cell clusters, the ClusterProfiler package [37] (v4.2.2) with default parameters was used for functional enrichment analyses, including GO, KEGG, and GSVA. Analyses focused on enrichments in signaling pathways and gene ontologies to reveal biological characteristics and functional differences in cell subsets. Results were primarily based on biological pathways from GO enrichment analysis, with specific pathways (e.g., endothelial proliferation, angiogenesis, and fibroblast activation) highlighted. Heatmaps of DEG expression patterns were generated using the R package pheatmap (v1.0.12). Bar plots with scatter overlays were created using ggplot2 [38] to show gene expression differences across groups and subsets.

### 2.5. Single Cell RNA Sequencing Data Analysis

ScRNA-seq data were downloaded from public repositories (GEO, China National Center for Bioinformation, and ArrayExpress) and integrated from four datasets: CRA007245 [39], E-MTAB-11940 [40], GSE213337 [41], and PRJNA1069235 [19]. The basic characteristics of these four single-cell RNA-seq datasets, including model type, modeling duration, mouse sex, and sequencing platform, are presented in Supplementary Table S4. Raw Fastq files were processed using CellRanger, including genome alignment, count filtering, barcode processing, and UMI counting. Filtered data were analyzed using the R package Seurat [42]. For quality control, cells with nFeature_RNA > 300, nFeature_RNA < 6000, and percent.mt < 15 were retained. Gene expression was normalized using “LogNormalize” with a scale factor of 10,000. ScaleData() was used to regress out effects of cell cycle, mitochondrial gene percentage, and UMI count. Principal component analysis (PCA) was performed using 2000 variable genes, and clustering was executed with FindClusters() and visualized via UMAP. Doublets were removed using DoubletFinder for each sample before integration [43]. Batch effects between datasets were corrected using the Harmony algorithm. Cell annotation was performed based on the CellMarker [44] database and published literature. DEGs between cell subsets were identified using FindAllMarkers, while DEGs between two groups were identified by FindMarkers, and ClusterProfiler was used for pathway and GO enrichment analyses.

### 2.6. Scissors Analysis

The scissors package [45] was used to integrate RNA sequencing and scRNA-seq data. T1D and T2D data were analyzed separately. A logistic regression model was used to determine relationships between cells and transcriptome sequencing data, identifying “scissors+” cells associated with diabetic phenotypes, which were then subjected to pathway enrichment analysis.

### 2.7. Statistical Analysis

Group comparisons for animal model data were performed using *t*-tests. For OGTT result, we calculated the AUC for each individual mouse to summarize the overall glucose tolerance, and compared the AUC values between groups using *t*-tests. Sequencing and scRNA-seq data analyses were conducted using R software (v4.2.3), with adjusted *p*-value < 0.05 being considered statistically significant. For DEG analysis, the false discovery rate (FDR) was controlled using the Benjamini–Hochberg method, with a significance threshold of FDR < 0.05. For pathway enrichment analysis, significant terms were defined using an FDR threshold of < 0.05 (also adjusted via the Benjamini–Hochberg procedure).

## 3. Result

### 3.1. Construction of Diabetic Models

**Figure 1:**
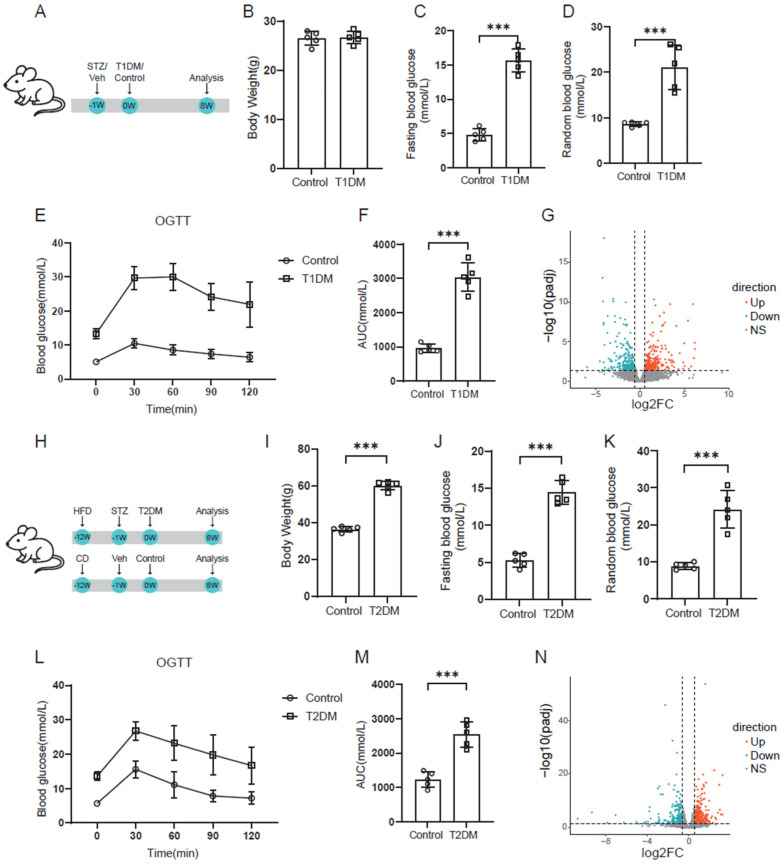
Model construction of T1D and T2D. (**A**) Schematic diagram of T1D modeling. (**B**) Body weight of T1D mice and control mice. (**C**,**D**) Fasting blood glucose and random blood glucose of T1D and control groups. (**E**) OGTT results of T1D and control groups. (**F**) Summary of the area under the curve (AUC) of OGTT results. (**G**) Volcano plot of differentially expressed genes between type 1 diabetic and control groups. (**H**) Schematic diagram of T2D modeling. (**I**) Body weight of T2D mice and control mice. (**J**,**K**) Fasting blood glucose and random blood glucose of T2D and control groups. (**L**) OGTT results of T2D and control groups. (**M**) Summary of the area under the curve (AUC) of OGTT results. (**N**) Volcano plot of differentially expressed genes between T2D and control groups. Each image of model construction is representative of 5 individual samples per group. ***: *p* < 0.001.

We established T1D and T2D mouse models in adult mice (Figure 1A,H) and compared their cardiac transcriptomes. Compared with control mice, T1D mice showed no significant changes in body weight (Figure 1B) but had markedly elevated fasting and random blood glucose levels (Figure 1C,D). OGTT revealed that glucose tolerance in T1D mice was significantly lower than that in the control group (Figure 1E,F). Further transcriptome sequencing of hearts from T1D mice and control mice identified 312 upregulated genes and 286 downregulated genes in T1D mice compared with controls (Figure 1G).

Similarly, the T2D mice displayed significantly higher body weight than the control mice (Figure 1I), with elevated fasting and random blood glucose levels (Figure 1J,K). OGTT also demonstrated impaired glucose tolerance in T2D mice (Figure 1L,M). Cardiac transcriptome analysis revealed 310 upregulated genes and 229 downregulated genes in T2D hearts relative to controls (Figure 1N).

### 3.2. Transcriptional Heterogeneity Between T1D and T2D

**Figure 2:**
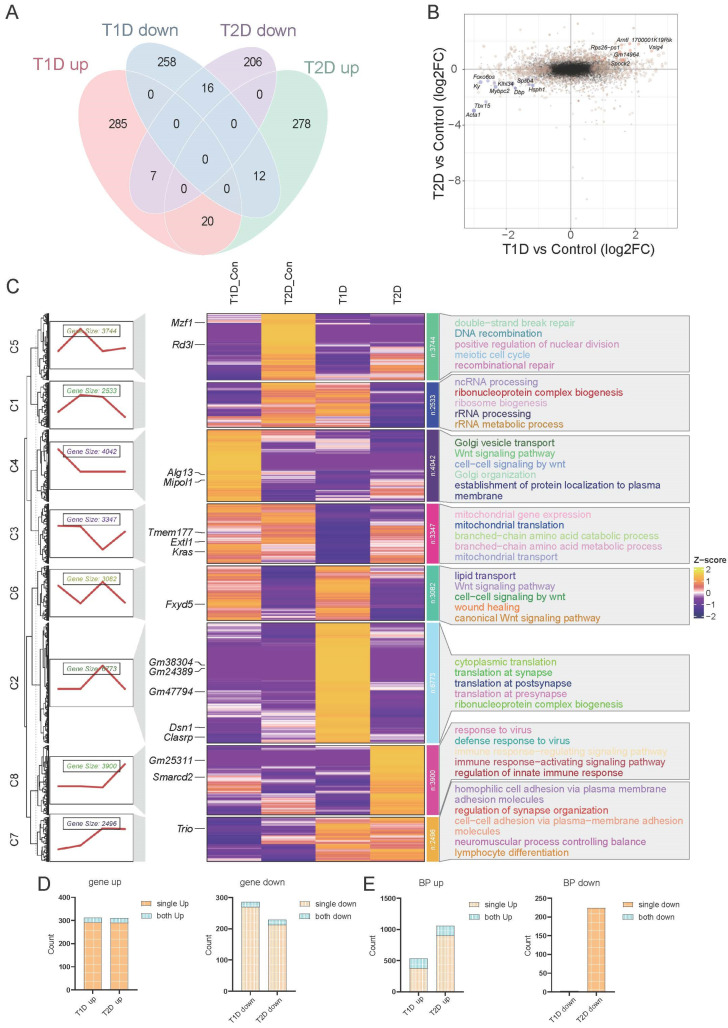
Clustering analysis of transcriptional profiles in T1D and T2D. (**A**) Intersection of up- and downregulated differentially expressed genes in the hearts of T1D and T2D mice. (**B**) Bidirectional volcano plot showing common changed genes in the hearts of T1D and T2D mice. Black dots represent genes with no differential expression. Light yellow dots indicate genes with significant fold changes between the diabetic group and the control group. Orange dots represent consistently and significantly upregulated genes, and blue dots represent consistently and significantly downregulated genes. (**C**) Clustering analysis of transcriptomic data from the hearts of T1D and T2D mice and their respective control groups. The line graph shows the gene expression patterns within each cluster. (**D**) The proportion of common differentially expressed genes in the hearts of T1D and T2D mice relative to their respective differentially expressed genes, with blue representing common differentially expressed genes and brown representing unique differentially expressed genes. (**E**) The proportion of common signaling pathways in the hearts of T1D and T2D mice relative to their respective signaling pathways, with blue representing common signaling pathways and brown representing unique signaling pathways.

We explored the similarities and differences in DEGs between T1D and T2D. Only 20 genes were upregulated and 16 genes were downregulated in both models (Figure 2A). By analyzing the expression changes of the two diabetic models, we found that genes showing identical expression patterns accounted for less than 10% (Figure 2B). GO enrichment analysis demonstrated that DEGs in T1D were significantly enriched in terms related to myocardial adaptive alterations such as myocardial hypertrophy (Supplementary Figure S1A), while DEGs in T2D were significantly enriched in terms such as innate immunity (Supplementary Figure S1B). KEGG enrichment analysis revealed that T1D was mainly enriched in retinol, phenylalanine, and tyrosine metabolism, as well as steroid hormone biosynthesis (Supplementary Figure S1C), while T2D was significantly enriched in immune-related diseases and antiviral responses (Supplementary Figure S1D).

Since conventional enrichment analysis is based on DEGs, we performed clustering analysis based on the overall gene expression profiles and divided genes into 8 clusters (Figure 2C). Genes in T1D were significantly clustered in cytoplasmic translation and ribonucleoprotein biosynthesis, while those in T2D were significantly clustered in antiviral and immune regulation (Figure 2C). Interestingly, T1D and T2D shared similar expression modules, which were significantly enriched in pathways including cell adhesion and lymphocyte differentiation (Figure 2C).

Based on the aforementioned results, we hypothesized that T1D and T2D may have common changes in signaling pathways, despite involving distinct gene sets. Therefore, we counted changes in genes and biological pathways in T1D and T2D. Consistent gene changes accounted for only 5–6% (Figure 2D), whereas the activated signaling pathways in the two models had a higher proportion of consistency (15–29% for upregulation, and 0% for downregulation; Figure 2E). Visualization of the conserved pathways further confirmed that T1D and T2D displayed concordant pathway alterations, with minimal overlap at the individual gene level (Supplementary Figure S1E,F).

### 3.3. Single-Cell RNA Sequencing Reveals Transcriptional Heterogeneity of Various Cell Types in Diabetes

#### 3.3.1. Landscape of Cardiac Cells in Diabetes

Bulk transcriptome sequencing fails to capture the adaptive changes of various cell types under pathological conditions. Therefore, we used scRNA-seq to analyze cellular differences in cells under different diabetic conditions. We integrated four publicly available scRNA-seq datasets, including data from T1D and T2D models. Following quality control and batch effect elimination, a total of 80,094 cells were retained for the subsequent analyses, among which 42,182 cells belonged to the control group, 5084 cells to the T1D group, and 32,828 cells to the T2D group.

**Figure 3:**
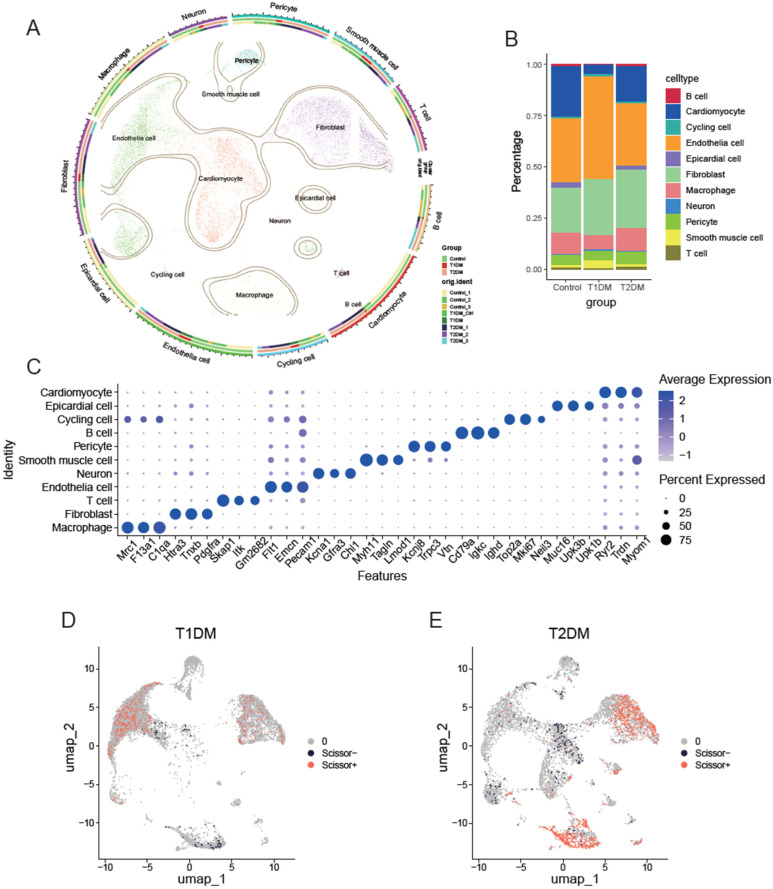
Single-cell RNA sequence of cardiac cells in T1D and T2D. (**A**) After quality control and Harmony batch correction, the sequencing data of T1D and T2D were dimensionally reduced and clustered into 11 distinct cell clusters, with clear differentiation between clusters. (**B**) Changes in the proportion of cell populations in T1D and T2D compared to controls. (**C**) Dot plots showing the top three marker genes for each cell cluster. (**D**) Scissors-identified cell populations associated with T1D phenotypes. (**E**) Scissors-identified cell populations associated with T2D phenotypes.

Dimensionality reduction and unsupervised clustering partitioned these cells into 11 distinct clusters, which were annotated according to marker genes from the CellMarker database (Figure 3A). Bar plots illustrating cellular proportions revealed that all major cell types were distributed across groups, supporting effective batch effect removal (Figure 3B). Combined with the expression patterns of signature genes in each subgroup, the cell clusters exhibited high discriminability (Figure 3C). Together, these results confirmed the reliable integration of scRNA-seq data and the clear identification of cell types for subsequent analyses.

We then applied the scissors algorithm to identify cell subsets highly correlated with diabetic phenotypes. By separately integrating RNA sequencing and scRNA-seq data for T1D and T2D, we found that endothelial cells and fibroblasts were the primary cell types significantly associated with the pathological phenotype in T1D (Figure 3D), while fibroblasts and macrophages were the key types in T2D (Figure 3E). We further analyzed endothelial cells, macrophages, and fibroblasts to explore their phenotypic differences between T1D and T2D.

#### 3.3.2. Endothelial Cells

**Figure 4:**
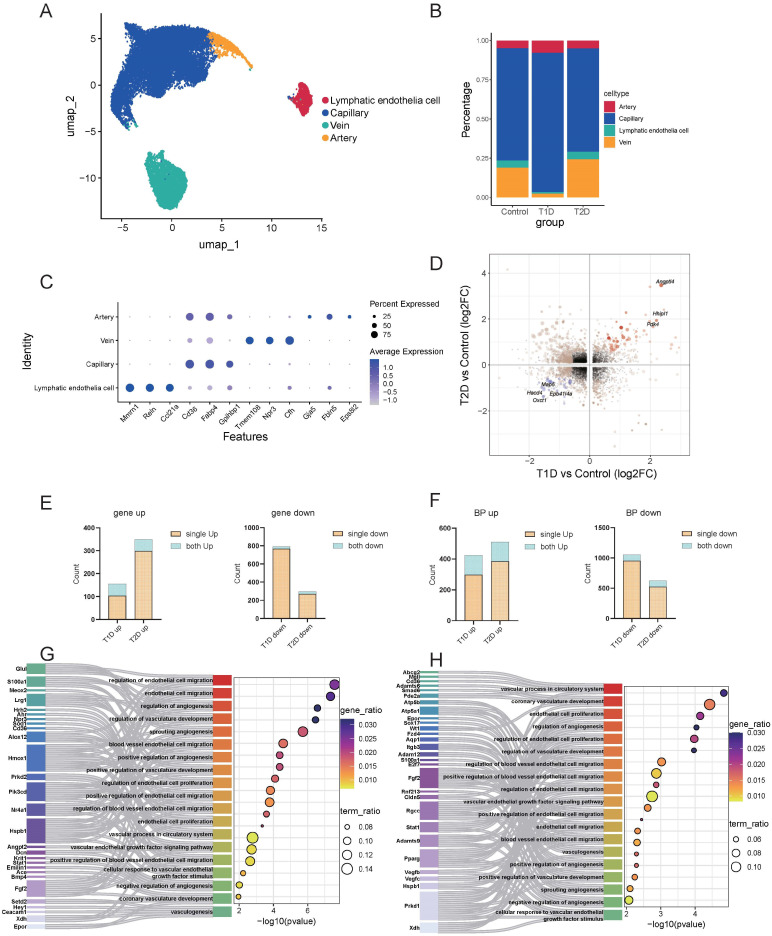
Transcriptional heterogeneity in endothelial cells under T1D and T2D. (**A**) Dimensionality reduction clustering of endothelial cells, categorized into lymphatic endothelial cells, capillary, vein, and artery. (**B**) Proportional changes of the four endothelial cell populations across groups. (**C**) Dot plots showing the expression levels of the top three marker genes for each cell cluster. (**D**) Bidirectional volcano plot showing common changed genes in the capillary endothelial cells of T1D and T2D. Black dots represent genes with no differential expression. Light yellow dots indicate genes with significant fold changes between the diabetic group and the control group. Orange dots represent consistently and significantly upregulated genes, and blue dots represent consistently and significantly downregulated genes. (**E**) The proportion of common differentially expressed genes in the capillary of T1D and T2D relative to their respective DEGs, with blue representing common DEGs and brown representing unique DEGs. (**F**) The proportion of common signaling pathways in the capillary of T1D and T2D relative to their respective signaling pathways, with blue representing common signaling pathways and brown representing unique signaling pathways. (**G**,**H**) Sankey diagrams showing the differences in common pathways in the capillary of T1D and T2D, illustrating the differences in DEGs across models.

Based on the aforementioned cell annotation results, we extracted endothelial cells and further subdivided them into four subgroups using previously reported cell markers [46]: capillary endothelial cells (capillary), lymphatic endothelial cells, venous endothelial cells (vein), and arterial endothelial cells (artery) (Figure 4A). All four subtypes were distributed across all experimental groups (Figure 4B) and exhibited distinct marker gene expression patterns (Figure 4C).

Enrichment analysis based on the transcriptional profiles of these four subtypes showed that lymphatic endothelial cells were significantly enriched in pathways including cell–matrix adhesion; capillary cells were enriched in pathways associated with cell migration and endothelial differentiation; vein cells were related to cardiac morphogenesis; and artery cells were linked to artery and organ development (Supplementary Figure S2A). Given that capillary endothelial cells showed the strongest association with endothelial function (Supplementary Figure S2B) and represented the most abundant subtype, we focused on capillary endothelial cells in subsequent analyses.

Comparative transcriptomic analysis of capillary between T1D and T2D identified few shared differentially expressed genes (Figure 4D). GO enrichment analysis demonstrated that the proportion of shared pathways between the two diabetic conditions (9–30%, Figure 4F) was higher than that of shared DEGs (3–33%, Figure 4E). T1D was significantly enriched in pathways related to inflammatory response, oxidative stress, and fatty acid metabolism (Supplementary Figure S2C), while T2D was enriched in mitochondrial and ribosomal functions, and energy metabolism (Supplementary Figure S2D). Visualization of shared endothelial function-related pathways in both diabetes types confirmed enrichment in endothelial proliferation, angiogenesis, and endothelial migration pathways, with few overlapping differential genes (Figure 4G,H).

#### 3.3.3. Macrophages

**Figure 5:**
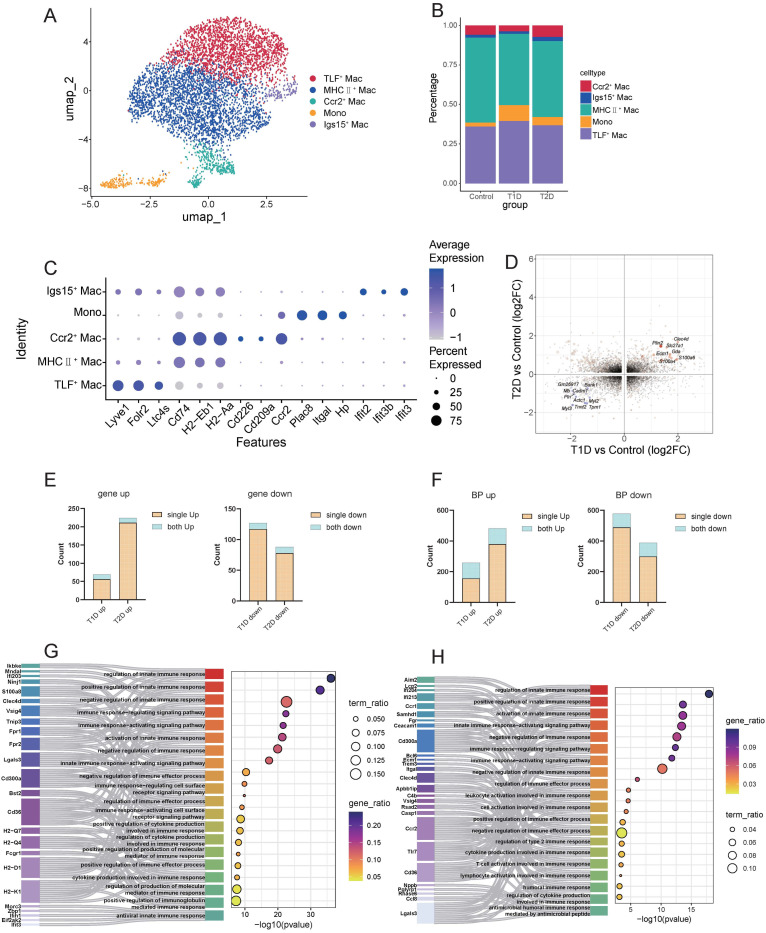
Transcriptional heterogeneity in macrophages under T1D and T2D. (**A**) Dimensionality reduction clustering of macrophages, categorized into TLR^+^ macrophages, MHCII^+^ macrophages, Ccr2^+^ macrophages, Igs15^+^ macrophages, and monocytes. (**B**) Proportional changes of macrophage populations across groups. (**C**) Dot plots showing the expression levels of the top three marker genes for each cell cluster. (**D**) Bidirectional volcano plot showing common changed genes in the macrophages of T1D and T2D. Black dots represent genes with no differential expression. Light yellow dots indicate genes with significant fold changes between the diabetic group and the control group. Orange dots represent consistently and significantly upregulated genes, and blue dots represent consistently and significantly downregulated genes. (**E**) The proportion of common DEGs in the macrophages of T1D and T2D relative to their respective DEGs, with blue representing common DEGs and brown representing unique DEGs. (**F**) The proportion of common signaling pathways in the macrophages of T1D and T2D relative to their respective signaling pathways, with blue representing common signaling pathways and brown representing unique signaling pathways. (**G**,**H**) Sankey diagrams showing the differences in common pathways in the macrophages of T1D and T2D, illustrating the differences in DEGs across models.

Macrophages were extracted from the integrated scRNA-seq dataset and further subdivided into five distinct subgroups using established marker genes: TLF^+^ macrophages, MHCII^+^ macrophages, Ccr2^+^ macrophages, Igs15^+^ macrophages, and monocytes (Figure 5A). Comparison of subgroup proportions showed increased monocyte infiltration in both diabetic models relative to the control group (Figure 5B), and all five subtypes showed clear discriminability based on their unique transcriptional profiles (Figure 5C).

Functional enrichment analysis demonstrated distinct functional specializations: TLF^+^ macrophages were associated with chemokine and leukocyte chemotaxis; MHCII^+^ macrophages were linked to tissue morphogenesis; Ccr2^+^ macrophages were enriched in ribosome biogenesis and transcription pathways; monocytes shared similar pathways with Ccr2^+^ macrophages and additionally participated in monocyte and lymphocyte proliferation; and Igs15^+^ macrophages were primarily associated with antiviral responses (Supplementary Figure S3A). Most subgroups were significantly enriched in immune-related pathways (Supplementary Figure S3B), consistent with the core functional role of macrophages in mediating immune responses.

Comparative transcriptomic analysis of macrophages between T1D and T2D revealed minimal overlap in differentially expressed genes (Figure 5D). Specifically, shared DEGs accounted for 5–18% (Figure 5E), while shared pathways accounted for 15–39% (Figure 5F). The top 30 pathways showed both diabetic types were enriched in pathways related to antigen presentation, pattern recognition receptors, antiviral responses, and immune responses in both types (Supplementary Figure S3C,D). Focusing on immune-related pathways, innate immune response pathways were activated in both diabetes types; but the underlying DEGs driving these pathway activations were significantly different between the two diabetic states (Figure 5G,H).

#### 3.3.4. Fibroblasts

**Figure 6:**
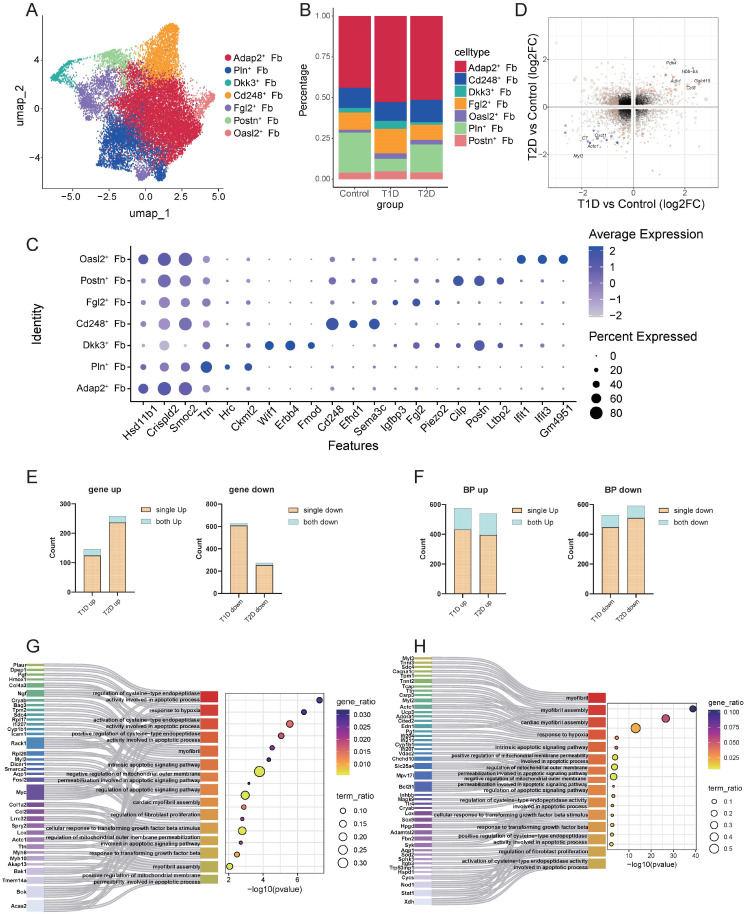
Transcriptional heterogeneity in fibroblasts under T1D and T2D. (**A**) Dimensionality reduction clustering of fibroblasts, categorized into 7 subpopulations of fibroblasts. (**B**) Proportional changes of fibroblast populations across groups. (**C**) Dot plots showing the expression levels of the top three marker genes for each cell cluster. (**D**) Bidirectional volcano plot showing common changed genes in the fibroblasts of T1D and T2D. Black dots represent genes with no differential expression. Light yellow dots indicate genes with significant fold changes between the diabetic group and the control group. Orange dots represent consistently and significantly upregulated genes, and blue dots represent consistently and significantly downregulated genes. (**E**) The proportion of common DEGs in the fibroblasts of T1D and T2D relative to their respective DEGs, with blue representing common DEGs and brown representing unique DEGs. (**F**) The proportion of common signaling pathways in the fibroblasts of T1D and T2D relative to their respective signaling pathways, with blue representing common signaling pathways and brown representing unique signaling pathways. (**G**,**H**) Sankey diagrams showing the differences in common pathways in the fibroblasts of T1D and T2D, illustrating the differences in DEGs across models.

Previous studies have highlighted the critical role of fibroblasts in the pathogenesis of DCM in both T1D and T2D. Our integrated analysis of bulk RNA sequence and scRNA-seq also confirmed their key role in diabetic phenotypes, prompting further exploration of their functions. Fibroblasts were subdivided into seven distinct subgroups using established marker genes: Adap2^+^ fibroblasts, Pln^+^ fibroblasts, Dkk3^+^ fibroblasts, Cd248^+^ fibroblasts, Fgl2^+^ fibroblasts, Postn^+^ fibroblasts, and Oasl2^+^ fibroblasts (Figure 6A). All subgroups were distributed across groups, with only mild changes in their proportions (Figure 6B), and exhibited distinct marker gene expression patterns (Figure 6C).

Functional enrichment analysis of each fibroblast subgroup revealed distinct functional profiles: the most abundant Adap2^+^ fibroblasts were significantly enriched in tissue/organ development and morphogenesis, suggesting that they may serve as a conserved subgroup. Pln^+^ fibroblasts were linked to energy metabolism; Dkk3^+^ fibroblasts were associated with connective tissue differentiation, particularly osteogenesis and calcification; Cd248^+^ fibroblasts were involved in vascular development and angiogenesis; Fgl2^+^ and Postn^+^ fibroblasts were enriched in extracellular matrix pathways; and Oasl2^+^ fibroblasts were linked to innate immunity and antiviral responses (Supplementary Figure S4A). Focusing on fibroblast-related and extracellular matrix pathways, Dkk3^+^, Fgl2^+^, and Postn^+^ fibroblasts were significantly enriched in pathways regulating fibroblast proliferation and functional activation (Supplementary Figure S4B).

Comparative analysis of DEGs between T1D or T2D and their matched control groups revealed minimal overlap (Figure 6D). Enrichment analysis demonstrated that the proportion of shared DEGs between the two types of diabetes was low (3–15%, Figure 6E), whereas the proportion of shared enriched ways was relatively higher (13–26%, Figure 6F). Specifically, T1D-associated fibroblasts were enriched in pathways related to innate immunity, translation, and antiviral responses (Supplementary Figure S4C), while T2D-associated fibroblasts were linked to cellular respiration and energy supply pathways (Supplementary Figure S4D). Visualization of shared fibroblast-related pathways confirmed that both diabetic types were enriched in hypoxia response, fibroblast proliferation, TGFβ, and apoptosis pathways in both diabetes types, despite minimal overlap at the DEG level (Figure 6G,H).

#### 3.3.5. Homogeneity of Cells in Different Types of Diabetes

**Figure 7:**
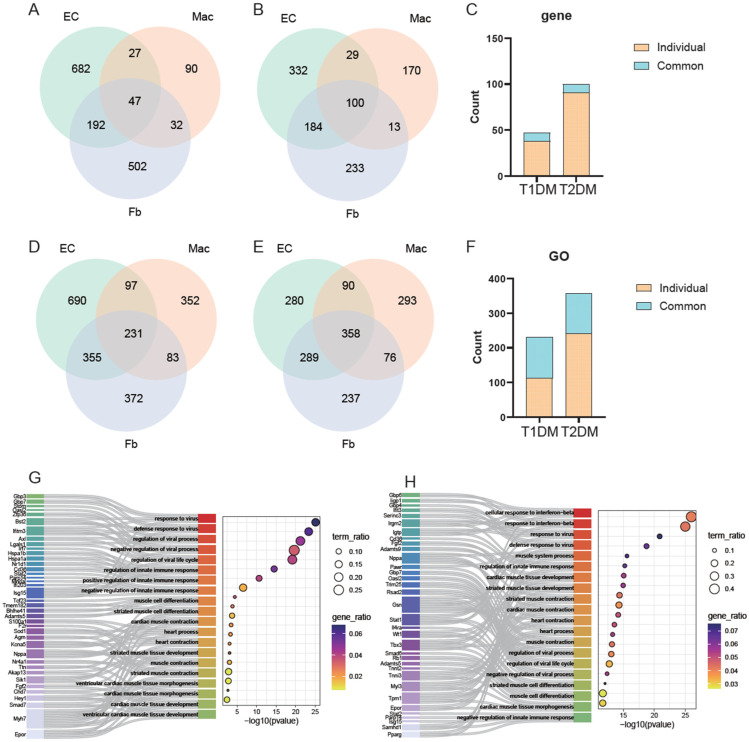
Common characteristics of different cell types in two types of diabetes. (**A**) Venn diagram of the intersection of DEGs in endothelial cells, macrophages, and fibroblasts in T1D. (**B**) Venn diagram of the intersection of DEGs in endothelial cells, macrophages, and fibroblasts in T2D. (**C**) The proportion of common DEGs in endothelial cells, macrophages, and fibroblasts in T1D and T2D relative to their respective DEGs, with blue representing common DEGs and brown representing unique DEGs. (**D**) Venn diagram of the intersection of signaling pathways in endothelial cells, macrophages, and fibroblasts in T1D. (**E**) Venn diagram of the intersection of signaling pathways in endothelial cells, macrophages, and fibroblasts in T2D. (**F**) The proportion of common signaling pathways in endothelial cells, macrophages, and fibroblasts in T1D and T2D relative to their respective signaling pathways, with blue representing common signaling pathways and brown representing unique signaling pathways. (**G**,**H**) Sankey diagrams showing the differences in common pathways in endothelial cells, macrophages, and fibroblasts in T1D and T2D, illustrating the differences in DEGs across models.

Given that both T1D and T2D are systemic diseases, we further explored whether they shared common gene or pathway changes across different cell types. The intersection analysis of DEGs in endothelial cells, macrophages, and fibroblasts identified 47 shared DEGs in T1D group (Figure 7A) and 100 shared DEGs in T2D group (Figure 7B). Notably, only nine genes were commonly shared between the two types diabetes, accounting for 10– 20% of the total shared DEGs in each disease (Figure 7C).

In terms of pathway enrichment, the three cell types shared 231 pathways in T1D and 358 in T2D (Figure 7D,E). The intersection analysis of these shared pathways betweenT1D and T2D yielded 117 pathways, accounting for 30–50% of the total shared pathways (Figure 7F). These results further reaffirmed that pathway changes are conserved between the two diabetes types, whereas the underlying DEGs driving these pathways show minimal overlap. Functional annotation of the common pathways shared among the three cell types in T1D and those in T2D indicated they primarily involved innate immune responses, antiviral responses, apoptosis, and cardiac morphogenesis (Supplementary Figure S5A,B). Top shared pathways, including antiviral/innate immune responses and cardiomyocyte function/differentiation, showed consistent enrichment in both diabetes types, yet with minimal overlap in the DEGs that regulate these pathways (Figure 7F,G).

## 4. Discussion

This study is based on transcriptome sequencing and scRNA-seq of cardiac tissues from T1D and T2D mice. Using multi-omics integrated data analysis, it elucidates the key pathophysiological alterations in cardiac remodeling in T1D and T2D, as well as pathway changes in different cell types during disease progression. By investigating and comparing the homogeneity and heterogeneity between t T1D and T2D cardiac remodeling, this work provides novel insights for future scientific research and clinical practice.

Through the integration of bulk RNA sequence and scRNA-seq, we identified endothelial cells, macrophages, and fibroblasts as key contributors to both types of diabetic cardiac remodeling. Most previous studies have focused on endothelial cell dysfunction and cell death [8,47]. In contrast, our study suggests that impaired proliferation of endothelial cells plays a critical role in this process. Consistent with previous reports in aorta [34], our study found that endothelial proliferation and angiogenesis pathways are enriched in both T1D and T2D mouse hearts, despite there being few overlapping genes between the two. Given our findings, the genes involved in endothelial proliferation exhibit almost no overlap between the two types of diabetes. Therefore, targeting the pathways and genes that regulate endothelial proliferation, such as the vascular endothelial growth factor (VEGF) or angiopoietin 1, may represent a promising therapeutic strategy for diabetic cardiac remodeling.

Additionally, the activation of immune functions in macrophages and endothelial cells leads to cytokine secretion, inducing programmed cell death, fibroblast activation and myocardial fibrosis, and further deteriorates cardiac remodeling [10]. Our results confirmed activated immune and inflammatory responses in macrophages and fibroblasts, promoting fibrotic processes in T1D and T2D mouse hearts. Prolonged hyperglycemia recruits and activates immune cells, triggering a cascade of inflammatory responses and leading to cardiac inflammation [48]. Cardiac inflammation, in turn, induces fibroblast proliferation, phenotypic transition, and collagen synthesis, while also impairing cardiomyocyte function and even promoting cardiomyocyte death [48,49,50]. Interestingly, our study revealed that not only macrophages, but also endothelial cells and fibroblasts participate in immune and inflammatory responses. We therefore propose that, in diabetes, multiple cell types collectively contribute to a microenvironment characterized by immune activation and inflammatory burst, which exacerbates cardiac stress and promotes the deterioration of cardiac function and remodeling. Future studies should integrate approaches such as spatial transcriptomics and metabolomics to define the local gene expression and metabolic characteristics during diabetic cardiac remodeling, thereby providing insights for the clinical management of diabetic cardiac complications.

This study has several limitations. Firstly, it is based on mouse models, which may have species-specific differences, and it lacks validation in human T1D and T2D data, restricting the generalizability of its conclusions. Secondly, while we integrated bulk RNA sequence and scRNA-seq to explore transcriptional differences, there is a lack of protein-level validation; future studies should incorporate proteomics analyses. Thirdly, the observed conservation of pathways in diabetic cardiac pathology suggests that dysfunction of transcriptional regulators may be a key area of focus, but current methods for comparing transcriptional factor activity across different diseases are limited, posing a technical challenge for future research. Last but not least, both diabetic mouse models employed in this study were established using streptozotocin (STZ), which is a well-established and widely used approach for inducing diabetes in preclinical research [51,52,53,54,55,56,57,58]. However, it should be noted that STZ may exert off-target systemic toxicity and direct effects on immune cell function and inflammatory signaling independent of hyperglycemia [59,60]. Therefore, the shared immune-related and inflammatory pathway signatures observed between the two models might reflect not only common biological alterations induced by hyperglycemia but also potential overlapping effects related to STZ-mediated cellular stress, tissue toxicity, and systemic perturbation. Future studies using non-STZ diabetic models, such as genetically obese insulin-resistant models or high-fat-diet-induced insulin resistance models, will be necessary to validate the immune-related mechanisms and clarify the specific contributions of hyperglycemia versus drug-induced effects.

Overall, our identification of conserved signaling pathways in the cardiac pathology of T1D and T2D provides new directions for both research and clinical applications. Targeting the common pathological pathway alterations shared by T1D and T2D, such as impaired endothelial proliferation, excessive immune activation, and inflammatory responses, may ameliorate cardiac remodeling and delay disease progression in both forms of diabetes.

## 5. Conclusions

This study constructed T1D and T2D models to investigate characteristic transcriptomic changes in diabetic cardiac remodeling. We found minimal overlap in DEGs between the two types diabetes but a high proportion of shared pathway changes. Integrating scRNA-seq with transcriptomic data, we identified endothelial cells, macrophages, and fibroblasts as significantly associated with diabetic phenotypes. Differential genes and enrichment analyses revealed greater consistency in pathway changes than gene changes in these three cell subsets under both diabetic conditions. Endothelial cells were significantly enriched in pathways related to endothelial proliferation, while macrophages were enriched in immune response pathways; and fibroblasts were enriched in pathways involving fibrosis. Finally, exploring commonalities across cell types highlighted significant changes in immune response and morphogenesis pathways in both diabetes types, suggesting their critical role in the development of cardiac remodeling.

## Data Availability

Data will be made available from the corresponding authors upon request.

## Supplementary Materials

The following supporting information can be downloaded at https://www.mdpi.com/article/10.3390/biomedicines14040746/s1. Figure S1: Enrichment Results of DEGs in the Hearts of T1D and T2D Mice, Figure S2: Enrichment Results of Cardiac Endothelial Cells in T1D and T2D, Figure S3: Enrichment Results of Macrophage in the Hearts of T1D and T2D Mice, Figure S4: Enrichment Results of Fibroblasts in the Hearts of T1D and T2D Mice, Figure S5: Enrichment Results of Common Signaling Pathways in the Hearts of T1D and T2D Mice, Table S1: Baseline characteristic of mouse model, Table S2: Formulation of High Fat Diet, Table S3: Quality Control of RNA sequence, Table S4: scRNA-seq datasets characteristics, Table S5: The top 10 marker genes for each cell type in scRNA-seq data.
