# Supplementary material for: Transcriptional Heterogeneity of Cardiac Remodeling Between Type 1 and Type 2 Diabetes
Source: Biomedicines. 2026 Mar 25;14(4):746. doi: 10.3390/biomedicines14040746 (PMC13114232; doi:10.3390/biomedicines14040746)
Supplement: Supplementary file 1 [file biomedicines-14-00746-s001.zip › biomedicines-4170593-supplementary.pdf]

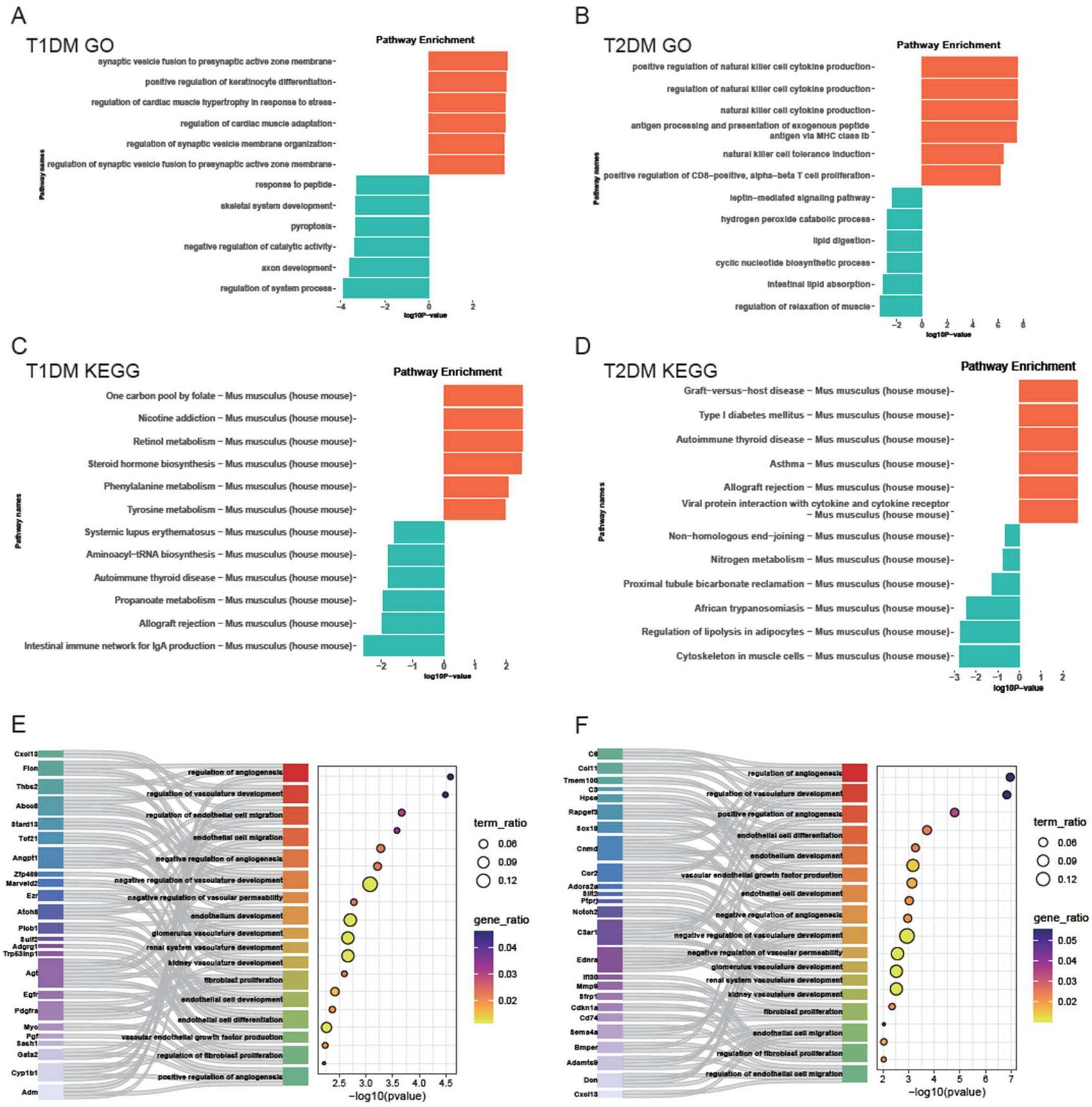

Sup Figure S1. Enrichment Results of DEGs in the Hearts of T1D and T2D Mice A: GO enrichment results of DEGs in T1D mice vs. controls; B: GO enrichment results of DEGs in T2D mice vs. controls; C: KEGG enrichment results of DEGs in T1D mice vs. controls; D: KEGG enrichment results of DEGs in T2D mice vs. controls; E, F: Sankey Diagrams showing common pathways in the hearts of T1D and T2D mice, illustrating the differences in DEGs across models.

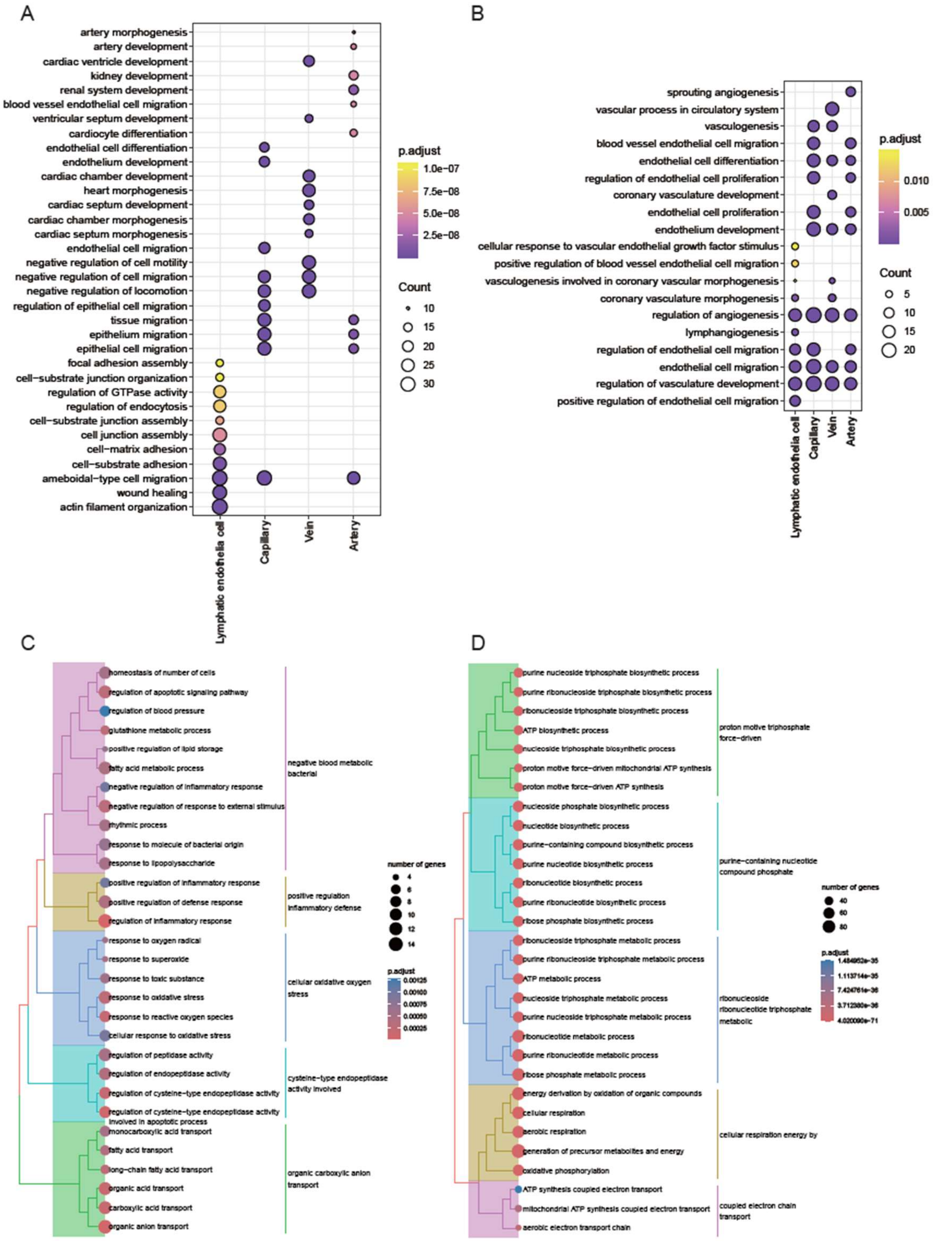

Sup Figure S2. Enrichment Results of Cardiac Endothelial Cells in T1D and T2D A: GO enrichment results of DEGs in different subpopulations of endothelial cells; B: Enrichment results of endothelial-related signaling pathways and angiogenesis pathways in different subpopulations; C: GO enrichment results of DEGs in capillary in T1D mice vs. controls; D: GO enrichment results of DEGs in capillary in T2D mice vs. controls.

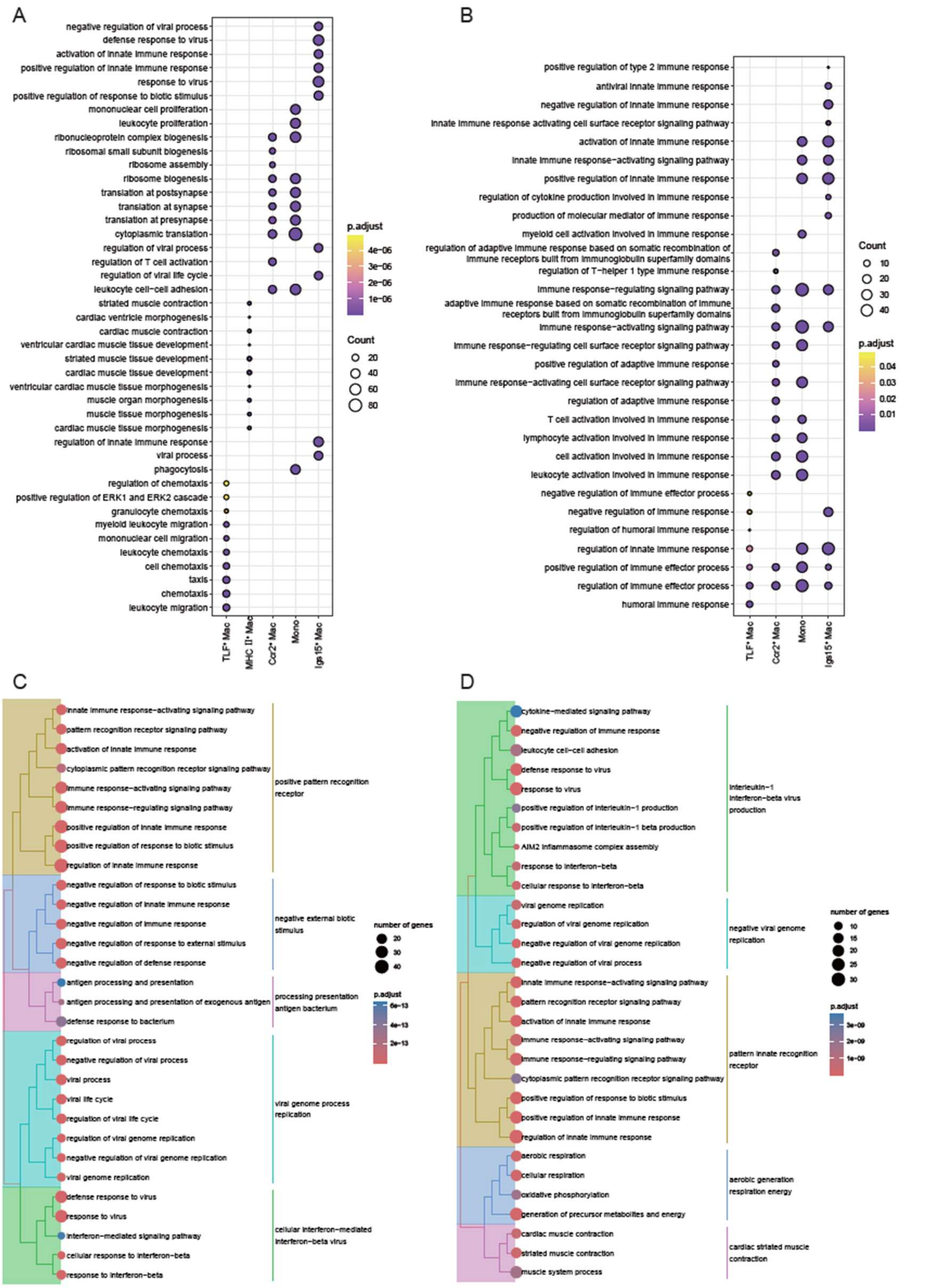

Sup Figure S3. Enrichment Results of Macrophage in the Hearts of T1D and T2D Mice A: GO enrichment results of DEGs in different subpopulations of macrophages; B: Enrichment results of immune-related pathways in different subpopulations; C: GO enrichment results of DEGs in macrophages in T1D mice vs. controls; D: GO enrichment results of DEGs in macrophages in T2D mice vs. controls.

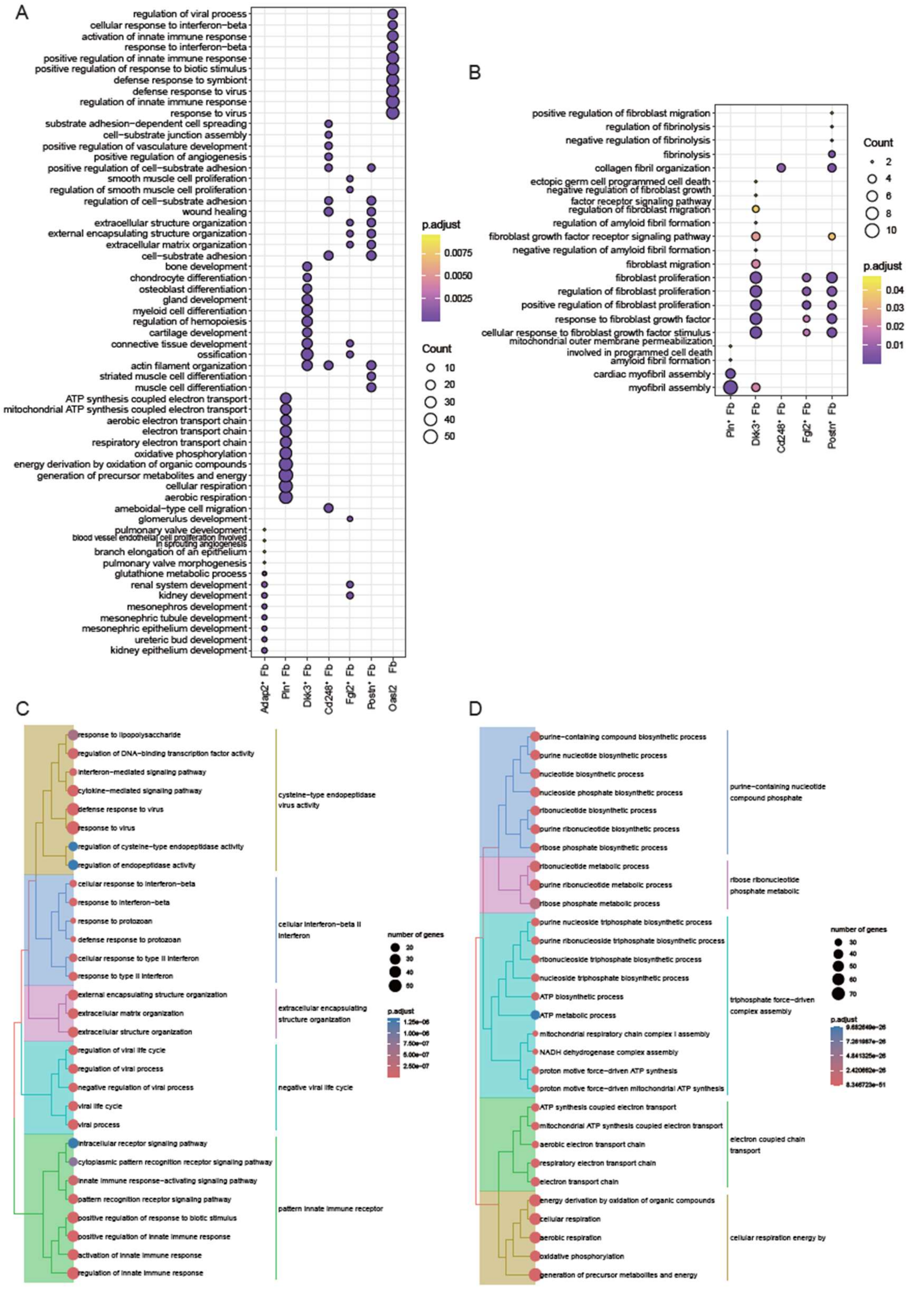

Sup Figure S4. Enrichment Results of Fibroblasts in the Hearts of T1D and T2D Mice A: GO enrichment results of DEGs in different subpopulations of fibroblasts; B: Enrichment results of fibroblast-related pathways and extracellular matrix pathways in different subpopulations; C: GO enrichment results of DEGs in fibroblasts in T1D mice vs. controls; D: GO enrichment results of DEGs in fibroblasts in T2D mice vs. controls.

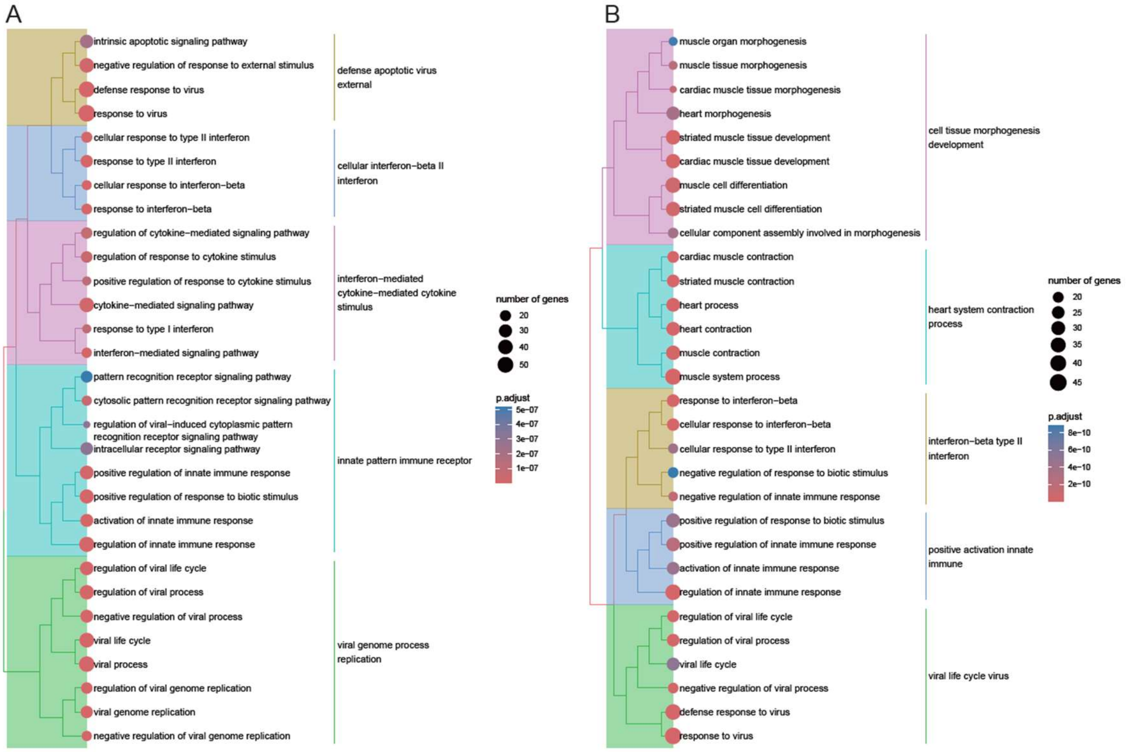

Sup Figure S5. Enrichment Results of Common Signaling Pathways in the Hearts of T1D and T2D Mice A: Enrichment analysis of common signaling pathways in endothelial cells, macrophages, and fibroblasts in T1D mice; B: Enrichment analysis of common signaling pathways in endothelial cells, macrophages, and fibroblasts in T2D mice.

Supplemetal Table S1: Baseline characteristic of mouse model.

T1D model baseline			
	Control	T1D	P value
Body weight: g	25.10±0.65	24.91±0.73	0.67
Fasting Glucose: mmol/L	4.98±0.57	4.80±0.56	0.62
T2D model baseline			
	Control	T2D	P value
Body weight: g	24.96±0.60	25.05±0.53	0.79
Fasting Glucose: mmol/L	4.88±0.53	4.96±0.70	0.84

Supplemetal Table S2: Formulation of High Fat Diet.

**Class description**	**Ingredients**	**Grams:g**	**kcal**
Protein	Casein, Lactic, 30 Mesh	200.00	800
Protein	Cystine, L	3.00	12
Carbohydrate	Lodex 10	125.00	500
Carbohydrate	Sucrose	72.80	275.2
Fiber	Solka Floc, FCC200	50.00	0
Fat	Lard	245.00	2205
Fat	Soybean Oil, USP	25.00	225
Mineral	S10026B	50.00	0
Vitamin	Choline Bitartrate	2.00	0

Vitamin	V10001C	1.00	40
Dye	Dye, Blue	0.05	0
Total		773.85	4057

Supplemetal Table S3: Quality Control of RNA sequence

Sample	RNA integrity metrics (RIN)	Library size	% Mapped reads	Error rate: %	Q30	GC%
T1D_Con_1	9.9	7.67G	95.0	0.01	94.64	46.65
T1D_Con_2	8.5	6.61G	88.93	0.01	95.23	42.56
T1D_Con_3	9.4	6.5G	94.83	0.01	95.13	46.64
T1D_Con_4	9.1	6.27G	93.82	0.01	94.71	45.92
T2D_Con_1	9.0	8.05G	93.31	0.01	94.56	44.62
T2D_Con_2	9.1	6.24G	93.91	0.01	94.65	45.82
T2D_Con_3	9.4	6.95G	94.68	0.01	95.44	45.55
T2D_Con_4	8.8	6.99G	93.95	0.01	94.47	44.75
T1D_1	9.2	5.96G	94.31	0.01	94.78	46.72
T1D_2	8.5	6.98G	91.97	0.01	94.90	46.27
T1D_3	8.9	6.99G	94.39	0.01	94.80	46.73
T1D_4	9.3	7.09G	94.02	0.01	94.83	46.43
T2D_1	8.7	6.88G	95.18	0.01	95.34	46.21
T2D_2	8.2	8.05G	89.56	0.01	96.92	44.45
T2D_3	8.8	7.08G	93.27	0.01	94.66	45.26
T2D_4	9.0	6.21G	94.73	0.01	94.92	45.56

Supplemetal Table S4: scRNA-seq datasets characteristics.

**Accession numbers**	**Diabetes model**	**Age**	**Duration**	**Sex**	**Tissue region**	**Sample number**	**Platform**
CRA007245	HFD/STZ -induced T2D	6W	21W	male	Whole heart	1 vs 1	Single Cell 3' V3 Reagent
E-MTAB-11940	spontaneously T2D (db/db)	17W	-	male	Cardiac ventricles	1 vs 1	Single Cell 3' V2 Reagent
GSE213337	STZ-induced T1D	8W	8W	male	Whole heart	1 vs 1	Single Cell 3' V3.1 Reagent
PRJNA1069235	spontaneously T2D (db/db)	12W	-	male	Whole heart	1 vs 1	Cell Ranger ARC v2.0.1

Supplemetal Table S5: The top 10 marker genes for each cell type in scRNA-seq data.

	p_val	avg_log2FC	pct.1	pct.2	p_val_adj	cluster	gene
1	0	6.82389295463417	0.399	0.005	0	Macrophage	F630028O10Rik
2	0	6.72758180655938	0.301	0.004	0	Macrophage	Ms4a4a
3	0	6.61141901659793	0.802	0.039	0	Macrophage	F13a1
4	0	6.55430413066606	0.382	0.006	0	Macrophage	Kcnk13
5	0	6.51039171934017	0.547	0.01	0	Macrophage	Aoah
6	0	6.47516008056267	0.708	0.014	0	Macrophage	Adgre1
7	0	6.46989326690774	0.477	0.008	0	Macrophage	Lilrb4a
8	0	6.45694558996994	0.429	0.007	0	Macrophage	Lilr4b
9	0	6.45080858778931	0.331	0.005	0	Macrophage	Tlr7
10	0	6.40234680545209	0.306	0.005	0	Macrophage	Cd33
11	0	4.4739655579192	0.703	0.08	0	Fibroblast	Clec3b
12	0	4.42186216059789	0.271	0.019	0	Fibroblast	Rgs17
13	0	4.36556357068516	0.351	0.028	0	Fibroblast	Hmcn2
14	0	4.35628562118748	0.734	0.08	0	Fibroblast	Dpep1
15	0	4.34231117554986	0.321	0.024	0	Fibroblast	Gfra1
16	0	4.31126758666685	0.361	0.029	0	Fibroblast	Bmper
17	0	4.30206748372961	0.389	0.032	0	Fibroblast	Svep1
18	0	4.29351095870445	0.987	0.538	0	Fibroblast	Gsn
19	0	4.28436179797944	0.795	0.086	0	Fibroblast	Htra3
20	0	4.28234516129161	0.563	0.045	0	Fibroblast	Scara5
21	0	10.4242050064546	0.621	0.002	0	T cell	Gm2682

22	0	10.1703406564327	0.65	0.001	0	T cell	Itk
23	0	9.93924245636199	0.403	0.005	0	T cell	Ccl5
24	0	9.81108649758189	0.464	0.002	0	T cell	Nkg7
25	0	9.67369795607188	0.469	0.001	0	T cell	Cd3e
26	0	9.67300046172689	0.574	0.002	0	T cell	Il7r
27	0	9.61272622621329	0.557	0.002	0	T cell	Cd3g
28	0	9.58719589924541	0.879	0.003	0	T cell	Skap1
29	0	9.57000568682554	0.422	0.002	0	T cell	Themis
30	0	9.32040498457769	0.454	0.001	0	T cell	Trbc1
31	0	3.68556183987068	0.263	0.051	0	Endothelia cell	Vwf
32	0	3.67534640833806	0.772	0.119	0	Endothelia cell	Emcn
33	0	3.63015027182131	0.335	0.041	0	Endothelia cell	Nos3
34	0	3.57123863343815	0.417	0.055	0	Endothelia cell	Rassf9
35	0	3.56359668992446	0.376	0.057	0	Endothelia cell	Samd12
36	0	3.53234065234468	0.544	0.078	0	Endothelia cell	Shank3
37	0	3.50963811254891	0.33	0.051	0	Endothelia cell	Cldn5
38	0	3.50131201511414	0.289	0.042	0	Endothelia cell	Car4
39	0	3.47679698304797	0.77	0.129	0	Endothelia cell	Cyyr1
40	0	3.46409198281059	0.447	0.061	0	Endothelia cell	Mmrn2
41	0	11.7730765391506	0.303	0	0	Neuron	Sostdc1
42	0	11.5481538154971	0.794	0.001	0	Neuron	Kcna1
43	0	11.398870767746	0.35	0	0	Neuron	Foxd3

44	0	11.3398236675509	0.625	0.001	0	Neuron	Gfra3
45	0	11.2025124287987	0.341	0	0	Neuron	Gjc3
46	0	11.0965755648383	0.494	0	0	Neuron	Sox10
47	0	11.0282386675415	0.494	0	0	Neuron	Gpr37l1
48	0	10.1887721948761	0.469	0.001	0	Neuron	Slitrk6
49	0	10.1545965733436	0.353	0	0	Neuron	Cmtm5
50	0	10.0076293608541	0.478	0.001	0	Neuron	Kcna6
51	0	9.00203677428871	0.394	0.001	0	Smooth muscle cell	Olfr78
52	0	8.42487622566356	0.802	0.03	0	Smooth muscle cell	Tagln
53	0	8.38791362415803	0.45	0.002	0	Smooth muscle cell	Map3k7cl
54	0	8.23651885601968	0.354	0.002	0	Smooth muscle cell	Cdh6
55	0	7.97986400137578	0.27	0.002	0	Smooth muscle cell	Cnn1
56	0	7.97523365643191	0.923	0.02	0	Smooth muscle cell	Myh11
57	0	7.83998963942642	0.914	0.051	0	Smooth muscle cell	Acta2
58	0	7.46563015176802	0.7	0.008	0	Smooth muscle cell	Lmod1
59	0	7.16294221449732	0.725	0.035	0	Smooth muscle cell	Myl9
60	0	7.05781090776924	0.504	0.005	0	Smooth muscle cell	Slc38a11
61	0	7.00173157200074	0.478	0.008	0	Pericyte	Lin7a
62	0	6.95155751790388	0.256	0.006	0	Pericyte	Zfp804b
63	0	6.77534244875524	0.701	0.021	0	Pericyte	Vtn
64	0	6.67456847765545	0.775	0.025	0	Pericyte	Trpc3
65	0	6.60041842848258	0.504	0.014	0	Pericyte	Mdga2

66	0	6.55518495957333	0.817	0.035	0	Pericyte	Kcnj8
67	0	6.52563643439099	0.475	0.012	0	Pericyte	Rerg
68	0	6.5094089725451	0.624	0.019	0	Pericyte	Pde8b
69	0	6.05903932951797	0.357	0.011	0	Pericyte	Nrxn1
70	0	6.00819660059386	0.378	0.012	0	Pericyte	Il34
71	0	10.371341558029	0.699	0.002	0	B cell	Pax5
72	0	10.3037868304264	0.721	0.002	0	B cell	Ms4a1
73	0	10.1455477203255	0.902	0.004	0	B cell	Cd79a
74	0	10.121788793573	0.25	0.001	0	B cell	Vpreb3
75	0	10.085792242727	0.306	0.001	0	B cell	Gm43388
76	0	10.0134563673613	0.35	0.001	0	B cell	Cd19
77	0	9.98096731127529	0.447	0.001	0	B cell	Gm30211
78	0	9.87284259155381	0.59	0.002	0	B cell	Fcmr
79	0	9.84190793982198	0.448	0.001	0	B cell	Pou2af1
80	0	9.82831990945371	0.67	0.003	0	B cell	Iglc2
81	0	10.026034933091	0.471	0.001	0	Cycling cell	Neil3
82	0	9.92902435370334	0.315	0	0	Cycling cell	Bub1
83	0	9.26182137641643	0.264	0	0	Cycling cell	Pimreg
84	0	9.04489594709034	0.298	0.001	0	Cycling cell	Depdc1a
85	0	8.95693432710187	0.31	0	0	Cycling cell	Pbk
86	0	8.74036757830616	0.377	0.001	0	Cycling cell	Hmmr
87	0	8.69363746964071	0.758	0.006	0	Cycling cell	Top2a

88	0	8.57651611233383	0.33	0.002	0	Cycling cell	Ube2c
89	0	8.54134468317868	0.342	0.001	0	Cycling cell	Esco2
90	0	8.47160233529425	0.293	0.001	0	Cycling cell	Melk
91	0	10.238348379718	0.402	0.001	0	Epicardial cell	Gm29266
92	0	9.30506274004106	0.741	0.004	0	Epicardial cell	Muc16
93	0	9.184404353	0.268	0	0	Epicardial cell	Vtcn1
94	0	8.87637164489874	0.398	0.001	0	Epicardial cell	Lrp2
95	0	8.75119817182455	0.32	0.001	0	Epicardial cell	Slc26a3
96	0	8.55861641971014	0.323	0.001	0	Epicardial cell	Chst4
97	0	8.47168991607182	0.621	0.003	0	Epicardial cell	Upk1b
98	0	8.30129242708967	0.616	0.009	0	Epicardial cell	Pcdh15
99	0	8.20605378431628	0.291	0.001	0	Epicardial cell	Bnc1
100	0	8.18218778145822	0.514	0.002	0	Epicardial cell	Lrrn4
101	0	5.17903902443061	0.301	0.014	0	Cardiomyocyte	Kcnn2
102	0	5.11669654797596	0.322	0.014	0	Cardiomyocyte	Gm31251
103	0	5.02700871674781	0.283	0.014	0	Cardiomyocyte	Hs3st5
104	0	4.93605548210448	0.449	0.043	0	Cardiomyocyte	Rbm20
105	0	4.89118757174534	0.397	0.031	0	Cardiomyocyte	D830005E20Rik
106	0	4.88636023156579	0.365	0.029	0	Cardiomyocyte	Grm1
107	0	4.87204994248199	0.267	0.013	0	Cardiomyocyte	D830032E09Rik
108	0	4.8283996911492	0.28	0.017	0	Cardiomyocyte	Gm35330
109	0	4.81638313157571	0.377	0.027	0	Cardiomyocyte	Tnni3k

110	0	4.65982336706024	0.312	0.018	0	Cardiomyocyte	Kbtbd12
